# Knockdown of Death-Associated Protein Expression Induces Global Transcriptome Changes in Proliferating and Differentiating Muscle Satellite Cells

**DOI:** 10.3389/fphys.2020.01036

**Published:** 2020-08-14

**Authors:** Katherine A. Horton, Kelly R. B. Sporer, Robert J. Tempelman, Yuwares Malila, Kent M. Reed, Sandra G. Velleman, Gale M. Strasburg

**Affiliations:** ^1^Department of Food Science and Human Nutrition, Michigan State University, East Lansing, MI, United States; ^2^Department of Animal Science, Michigan State University, East Lansing, MI, United States; ^3^National Center for Genetic Engineering and Biotechnology (BIOTEC), Thailand Science Park, Pathum Thani, Thailand; ^4^Department of Veterinary and Biomedical Sciences, University of Minnesota, Saint Paul, MN, United States; ^5^Department of Animal Sciences, Ohio Agricultural Research and Development Center, The Ohio State University, Wooster, OH, United States

**Keywords:** satellite cells, muscle development, death-associated protein, global gene expression, microarray, muscle, turkey

## Abstract

Death-associated protein (DAP) undergoes substantial changes in expression during turkey skeletal muscle development, decreasing from the 18 day embryonic stage to 1 day posthatch, and again from 1 day posthatch to 16 weeks of age. These changes suggest that DAP plays an important role at critical stages of the developmental process. The objective of this study was to elucidate the role of DAP in muscle development by examining the effect of reduced *DAP* expression on global gene expression in proliferating and differentiating turkey *pectoralis major* muscle satellite cells. Small interfering RNA was used to knock down expression of *DAP* and the transcriptome was subsequently profiled using a turkey skeletal muscle long oligonucleotide microarray. Microarray data were corroborated using quantitative real-time PCR. In proliferating cells, 458 loci, resulting in 378 uniquely annotated genes, showed differential expression (false discovery rate, FDR < 0.05). Pathway analysis highlighted altered eukaryotic translational initiation factors (eIFs) signaling, protein ubiquitination, sirtuin signaling, and mechanistic target of rapamycin (mTOR) signaling as the primary pathways affected in the knockdown proliferating cells. The findings underpinned the potential DAP involvement in cell proliferation of turkey satellite cells through the coordination between protein synthesis and cell cycle. In differentiating cells, 270 loci, accounting for 189 unique genes, showed differential expression (FDR < 0.05). Decreased expression of genes encoding various myofibrillar proteins and proteins involved in sarcoplasmic reticulum calcium flux suggests that DAP may affect regulation of calcium homeostasis and cytoskeleton signaling. This study provides the first evidence that reduced expression of *DAP* significantly alters the transcriptome profile of *pectoralis major* muscle satellite cells, thereby reducing proliferation and differentiation.

## Introduction

Death-associated protein (DAP) is a highly conserved, 15-kDa, proline-rich protein with two phosphorylation sites (Deiss et al., 1995; Koren et al., 2010). It was first identified by Deiss et al. (1995) in studies aimed at identifying novel genes with functional relevance in apoptosis. Subsequent cell culture studies found that in nutrient-rich conditions, DAP is a substrate for phosphorylation by the mechanistic target of rapamycin (mTOR) (Koren et al., 2010). When cells are subjected to amino acid starvation, mTOR activity is down-regulated and DAP undergoes dephosphorylation. Analysis of cells grown under nutrient-rich and nutrient-deprived conditions suggests that the phosphorylated form is functionally silent, whereas dephosphorylated DAP is the active form that acts as an inhibitor of cell autophagy (Koren et al., 2010). Subsequently, others have shown that DAP is involved in regulation of autophagy and apoptosis induced by subtilase cytotoxin (SubAB) of some strains of Shiga-toxigenic *E. coli* (Yahiro et al., 2014). Reduced expression of *DAP* is also associated with adverse clinical outcomes for human breast (Wazir et al., 2012) and colorectal cancer (Jia et al., 2014) owing to altered regulation of apoptosis and autophagy.

In previous studies, our group investigated changes in gene expression in turkey *pectoralis major* muscle as a function of temporal development (Sporer et al., 2011b). Based on the following criteria, *DAP* was one of several differentially expressed (DE) genes selected for further studies. First, *DAP* expression changed substantially, being highest in embryonic muscle undergoing hyperplasia, followed by a five-fold decrease in neonatal muscle undergoing hypertrophy, and decreased further in muscle from 16-wk market-age turkeys (Sporer et al., 2011b). Second, little was known about the function of DAP, and most importantly, there was no previous evidence for its role in muscle development.

In subsequent studies, knockdown of *DAP* expression in turkey *pectoralis major* muscle satellite cells by small interfering RNA (siRNA) resulted in reduction of proliferation by up to 50% and nearly complete inhibition of the differentiation of satellite cells into myotubes (Velleman et al., 2012). Chicken satellite cells transfected with *DAP* cDNA showed that overexpression of *DAP* resulted in increased proliferation, differentiation, and myotube diameter, but had no effect on satellite cell apoptosis (Shin et al., 2013a). These studies were the first to suggest that DAP may play an important role in *pectoralis major* muscle development.

The objective of the current study was to initiate studies to define the role of DAP in the early stages of muscle growth and development by testing the hypothesis that altered *DAP* expression modulates expression of other genes that are critical to *pectoralis major* muscle development. siRNA was used to reduce *DAP* expression in proliferating and differentiating turkey myogenic satellite cells. Transcriptome differences between control and knockdown cell cultures were measured using a turkey skeletal muscle long oligonucleotide microarray (TSKLMO) (Sporer et al., 2011a). Our results provide the first evidence of global gene expression changes upon knockdown of *DAP* expression. Pathway analysis suggests potential involvement of *DAP* in protein synthesis, calcium homeostasis, and muscle mass accumulation.

## Materials and Methods

### Isolation of Turkey Myogenic Satellite Cells

Randombred Control Line 2 (RBC2) *Pectoralis major* muscle satellite cells, previously harvested according to McFarland et al. (1988) and stored in liquid nitrogen, were used for this study. The RBC2 line represents a 1967 commercial line turkey that has been maintained at The Ohio State University, Agricultural Research and Development Center Poultry Research Unit, without conscious selection for any traits (Nestor et al., 1969; Nestor, 1977). An ethics approval statement was not required because no animals were sacrificed for the current study. The cells were analyzed for proliferation and differentiation characteristics as previously described (Velleman et al., 2000, 2012).

### Cell Culture

**FIGURE 1:**
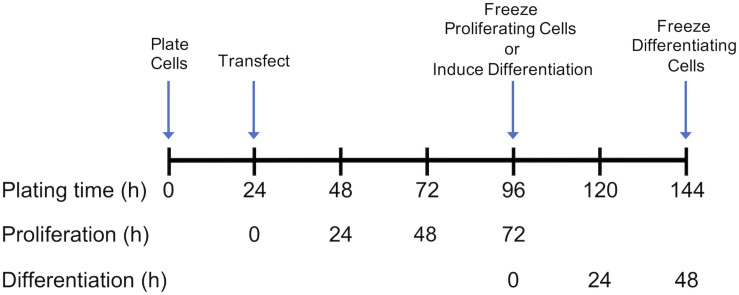
Timeline for plating, transfection, proliferation, and differentiation of satellite cells. Following transfection or mock transfection, cells underwent proliferation for 72 h and were frozen for subsequent analysis. Alternatively, the medium was changed at 72 h to induce differentiation. Following 48 h of differentiation, cells were frozen for subsequent analysis. Experimental details are provided by Velleman et al. (2012).

Growth of control and *DAP*-knockdown satellite cells were performed at The Ohio State University as described in detail by Velleman et al. (2012). Briefly, satellite cells were transfected with siRNA targeted against *DAP* or were mock-transfected (without siRNA) 24 h after plating (Figure 1). Proliferation was induced as previously described (Velleman et al., 2006, 2012) and the extent of proliferation was measured by DNA concentration (McFarland et al., 1995). After 72 h of proliferation, cell differentiation was induced by changes in growth media as described by Velleman et al. (2012) (Figure 1), The differentiation assay was adapted from Florini (1989) and Yun et al. (1997) as described by Velleman et al. (2012). The extent of differentiation was determined as a function of muscle-specific creatine kinase levels at 48 h after induction of differentiation.

### RNA Isolation

Satellite cell cultures were frozen at −80°C after 72 h of proliferation or 48 h of differentiation and shipped on dry ice to Michigan State University for RNA isolation and subsequent microarray hybridization. Frozen cells were thawed on ice for 5 min prior to isolation of total RNA with TRIReagent (Molecular Research Center, Inc., Cincinnati, OH, United States) following manufacturer instructions. Contaminating genomic DNA was removed by treatment with RNA-free DNase (Ambion Inc., Austin, TX, United States). Total RNA was quantified using a Nanodrop ND-1000 spectrophotometer (Thermo Fisher Scientific, Waltham, MA, United States) and sample integrity determined with an Agilent 2100 Bioanalyzer (Santa Clara, CA, United States). Samples with an RNA Integrity Number (RIN) of >8.0 were considered acceptable for use with the microarray.

### Microarray Experimental Design

Design, construction, and validation of the TSKMLO array are described in Sporer et al. (2011a). The microarray was constructed based on cDNA libraries gathered at critical stages in turkey skeletal muscle development (Reed et al., 2008). Information regarding the TSKMLO platform can be found at the National Center for Biotechnology (NCBI) Gene Expression Omnibus (GEO) database (platform accession: GPL9788). In the current study, RNAs isolated from *DAP* knockdown samples were directly compared to those of the control treatment. A total of four replicates was used for each experiment (proliferation or differentiation) and dye swaps were included in the experimental design to minimize dye bias, resulting in eight arrays per experiment and 16 arrays for the entire knockdown study.

### RNA Amplification and Microarray Hybridization

Total RNA was amplified, dye-coupled, and purified using the Amino Allyl MessageAmp^TM^ II aRNA Amplification Kit (Ambion, Inc.) according to the manufacturer’s protocol. Due to low levels of RNA isolated from cell culture samples, two rounds of amplification were performed for all samples. Microarray preparation and hybridization were carried out as described in Sporer et al. (2011a). All spot intensities were exported as GenePix Results (GPR) files for statistical analysis.

### Microarray Statistical Analysis and Gene Annotation

The GPR files were subjected to statistical analysis as described in Sporer et al. (2011b). Briefly, dye intensity bias was normalized using the “normexp” background correction method based on Ritchie et al. (2007). Normalized data were described as log_2_ fluorescent intensities ratio (Cy5/Cy3) or *M*-value, and statistically analyzed with a linear model using LIMMA (Smyth, 2005) based on overall intercept, the dye assignment for samples from the *DAP* knockdown treatment, and the random effect of cells derived from the treatment. Mismatch and negative control spots on the microarray were used to confirm that hybridization to the array was specific (Sporer et al., 2011a). Raw Cy5 and Cy3 intensities, *M*-value, and LOESS-normalized log_2_ average intensities (A) were submitted with original GPR files to the NCBI GEO (platform accession: GPL9788, series accession: GSE35660).

Differences in expression levels between *DAP* knockdown and control were considered significant with an estimated false discovery rate (FDR) <0.05. In this experiment, the comparison of interest was overall differences between *DAP* knockdown treatment and control within each developmental stage (proliferation or differentiation). Fold change was defined as the ratio of expression of particular gene in *DAP*-knockdown cells relative to control cell culture. Gene annotation of the array probes was conducted by iterative NCBI BLAST searches (blast.ncbi.nlm.nih.gov) of the original cDNA sequences used to design the array probes. Sequences were first queried against the turkey refseq gene list (genome annotation 103), then the NCBI refseq database, followed by the turkey genome (v5.1) and the NCBI NR database. Sequences that failed to identify significant matches were designated as novel transcripts. Several sequences aligned to introns within described genes in the turkey genome BLAST searches. These were assigned to the described genes. Sequences with significant alignment to the turkey genome outside of described genes and without matches to other databases were designated as undescribed transcripts.

### Confirmation of Gene Expression by Quantitative Real-Time PCR

Six genes from the proliferation experiment and 9 genes from the differentiation experiment were chosen for gene expression analysis using quantitative real-time PCR (qPCR). Genes were chosen based on function and comparably high levels of differential expression as seen in the microarray analysis. Primers (Supplementary Table S1) were designed using Primer Express 3.0 software (Applied Biosystems, Foster City, CA, United States) and synthesized by Operon, Inc. (Huntsville, AL, United States). Sample Amino Allyl-aRNAs (2 μg), prepared for microarray hybridization using the Amino Allyl MessageAmp^TM^ II aRNA Amplification Kit (Ambion, Inc.), were reverse-transcribed into cDNA using Superscript III (Invitrogen). Synthesized cDNA was quantified using a Nanodrop. Reactions were run using 6–10 ng cDNA template, 1.2 μM primer mix, and Power SYBR Green PCR Master Mix (Applied Biosystems) in an ABI Prism 7900HT Sequence Detection System (Applied Biosystems). Data were analyzed using the 2^–ΔΔCt^ method (Livak and Schmittgen, 2001). Based on data indicating no change in C_t_ for the described treatment conditions (data not shown), UNC-5 homolog B (UNC5B) and Integral Membrane Protein 2C (IMP2C) were used as endogenous control genes for proliferation and differentiation, respectively. Statistical analysis was performed using a *t*-test (SigmaPlot, San Jose, CA, United States) and results are expressed as a fold change of the *DAP* knockdown relative to the control.

### Functional Analysis

DE transcripts (FDR < 0.05) were subjected to pathway analysis using Qiagen Ingenuity Pathway Analysis (IPA) software (Qiagen, Redwood City, CA, United States). Canonical pathways, indicative of cell signaling and metabolic pathways relevant to the DE genes, were obtained from IPA analysis based on algorithms developed by Krämer et al. (2014).

## Results

### Differential Expression of Satellite Cell Genes

**FIGURE 2:**
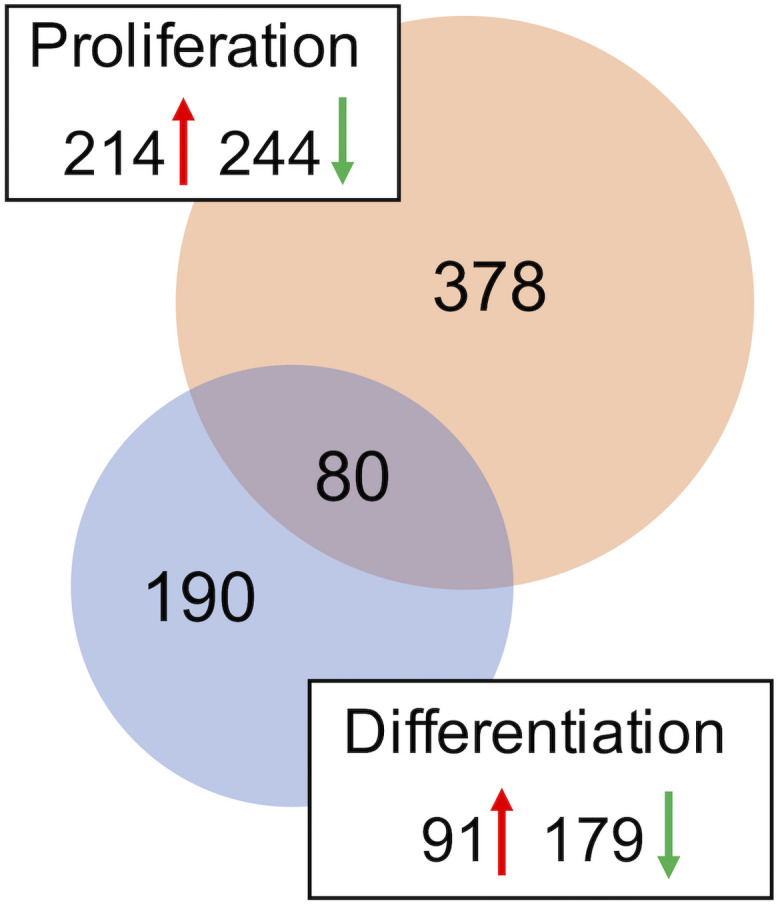
Distribution of differentially expressed genes in the turkey satellite cells. For each comparison, the number of significant genes (FDR *p*-value < 0.05) shared or unique to each treatment are indicated in the Venn diagram. Circle size is proportional to the number of genes and direction of expression change (↑ or ↓) is given for each group.

A combined total of 647 loci (representing up to 559 genes) was DE between *DAP* knockdown and control treatments in the turkey satellite cells (FDR < 0.05). In the proliferating cells, 458 loci (378 unique genes) were DE (Supplementary Table S2), whereas 270 loci (189 unique genes) were DE in differentiation (Figure 2). During proliferation, more genes were down-regulated (244) than up-regulated (214) upon knockdown of *DAP* (Supplementary Table S2).

As with knockdown of proliferating satellite cells, more genes in the differentiating satellite cells were down-regulated (179) than up-regulated (91) (Supplementary Table S3). As expected, expression of most of the myofibrillar proteins were decreased. Those include two loci of alpha actin (*ACTA1*, FC = −19 and −12), various isoforms of tropomyosin (*TPM*, FC ranging from −4 to −15), troponin C (*TNNC*, FC ranging between −2 and −18), troponin I (*TNNI*, FC ranging from −2.6 to −13), troponin T (*TNNT*, FC ranging from −1.9 to −35), nebulin (*LOC109368623*, FC = −4), and titin (*TTN*, FC = −3.5). In addition, the gene for obscurin (*OBSCN*), a protein linking the myofibril to the sarcoplasmic reticulum (SR), was down-reglated 2.2-fold.

Eighty loci showed differential expression in both proliferation and differentiation knockdown experiments (Table 1). Expression of 72 loci was directionally consistent between both the proliferating and differentiating cells. Differences in fold change between proliferation and differentiation ranged from 0.009 to 23.9 with an average of 2.6 (Standard Deviation = 3.61). The largest FC deviation was observed for eukaryotic translation initiation factor 3 subunit E (*eIF3E*) with FC = 29.3 and 5.3 in proliferation and differentiation, respectively. This initiation factor is a component of the initiation factor 3 (eIF-3) complex, required in the initiation of protein synthesis. In contrast, FC values were essentially identical for *CDV3* (2.37 vs 2.36). Protein CDV3 homolog, also known as carnitine deficiency-associated gene expressed in ventricle 3, encodes the protein Histone H4.

**TABLE 1:** Differentially expressed transcripts identified in both proliferation and differentiation knockdown experiments.

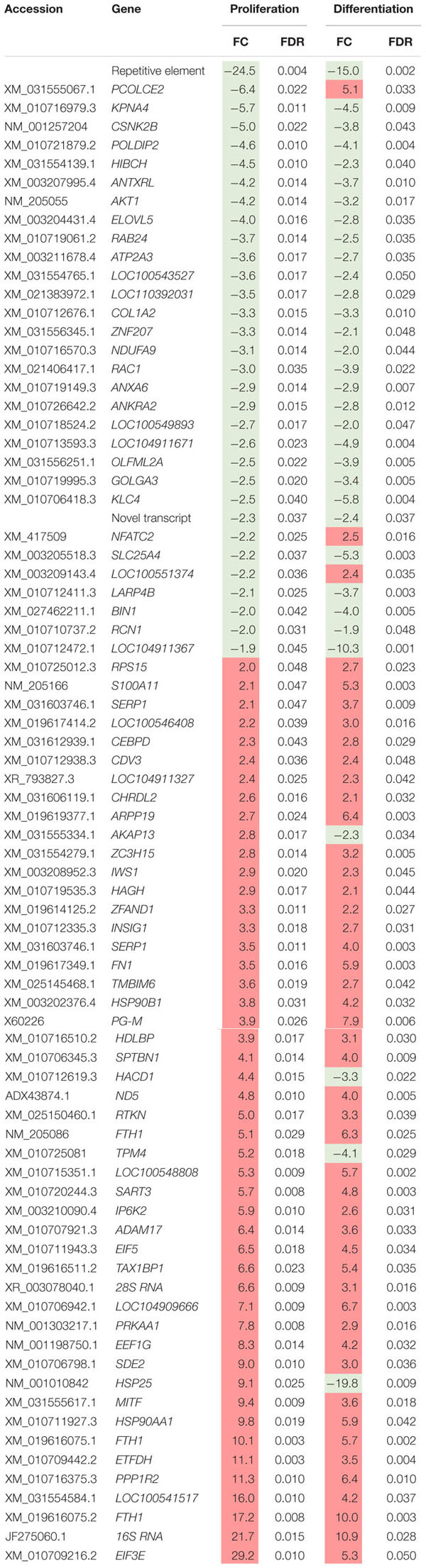

*FC, fold change, positive and negative FC indicates increased and decreased, respectively, abundance in *DAP*-knockdown satellite cells. FDR, false discovery rate. Green highlighting indicates down-regulated genes; red highlighting indicates up-regulated genes.*

Four gene loci that were up-regulated during proliferation were down-regulated in differentiation (Table 1). These loci include heat shock protein 25 (*HSP25*, FC = 9.1 vs −19.8), tropomyosin 4 (*TPM4*, FC = 5.2 vs −4.1), 3-hydroxyacyl-CoA dehydratase 1 (*HACD1*, FC = 4.3 vs −3.3), and A-kinase anchoring protein 13 (*AKAP13*, FC = 2.8 vs −2.2). Conversely, three gene loci that were down-regulated during proliferation were up-regulated in differentiation. These include deoxyribodipyrimidine photo-lyase-like (*LOC100551374*, FC = −2.19 vs 2.35), nuclear factor of activated T-cells (*NFATC2*, FC = −2.20 vs 2.53), and procollagen C-endopeptidase enhancer 2 (*PCOLCE2*, FC = −6.4 vs 5.1).

### Confirmation of Gene Expression

**FIGURE 3:**
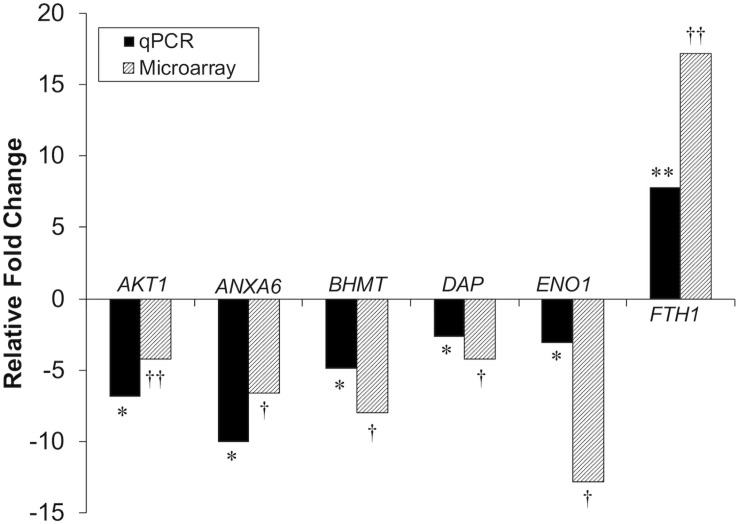
Microarray and qPCR data show similar trends of gene expression in proliferation experiments. Fold change is defined as the change in gene expression in the *DAP* knockdown samples relative to control samples. Bars below the origin indicate lower expression (down-regulation) of the gene in the *DAP* knockdown samples; bars above the origin indicate higher expression (up-regulation). *P < 0.05, **P < 0.01, ^†^FDR < 0.05, and ^††^FDR < 0.01. *AKT1*, Akt1; *ANXA6*, Annexin A6; *BHMT*, Betaine-homosysteine *S*-methyltransferase; *DAP*, death-associated protein; *ENO1*, enolase 1; *FTH1*, Ferritin heavy polypeptide 1.

**FIGURE 4:**
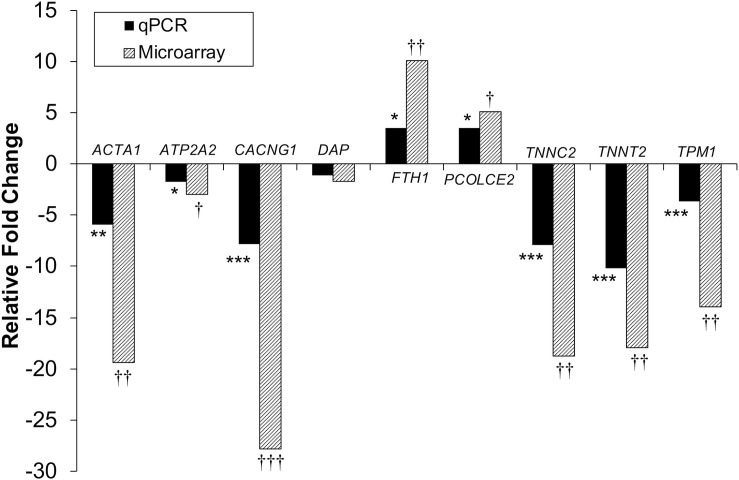
Microarray and qPCR data show similar trends of expression differences in differentiation experiments. Fold change is described as change in gene expression in the *DAP* knockdown samples relative to controls. Bars below the origin indicate lower expression (down-regulation) of the gene in the *DAP* knockdown samples; bars above the origin indicate higher expression (up-regulation). *P < 0.05, **P < 0.01, ***P < 0.001, ^†^FDR < 0.05, ^††^FDR < 0.01, and ^†††^FDR < 0.001. *ACTA1*, actin alpha 1 skeletal; *ATP2A2*, sarcoplasmic/endoplasmic reticulum ATPase 2 cardiac; *CACNG1*, voltage dependent calcium channel gamma 1; *DAP*, death-associated protein; *FTH1*, ferritin heavy polypeptide 1; *PCOLCE2*, procollagen C-endopeptidase enhancer; *TNNC2*, Troponin C type 2 fast skeletal; *TNNT2*, Troponin T type 2 cardiac; *TPM1*, Tropomyosin 1.

Differential gene expression identified by microarray analysis was corroborated by performing qPCR on six genes from the proliferation experiment and nine genes from the differentiation experiment (Supplementary Table S1). All genes selected show similar overall trends in differential expression in both microarray and in qPCR results for proliferation (Figure 3) and differentiation (Figure 4).

### Functional Pathway Analysis

**FIGURE 5:**
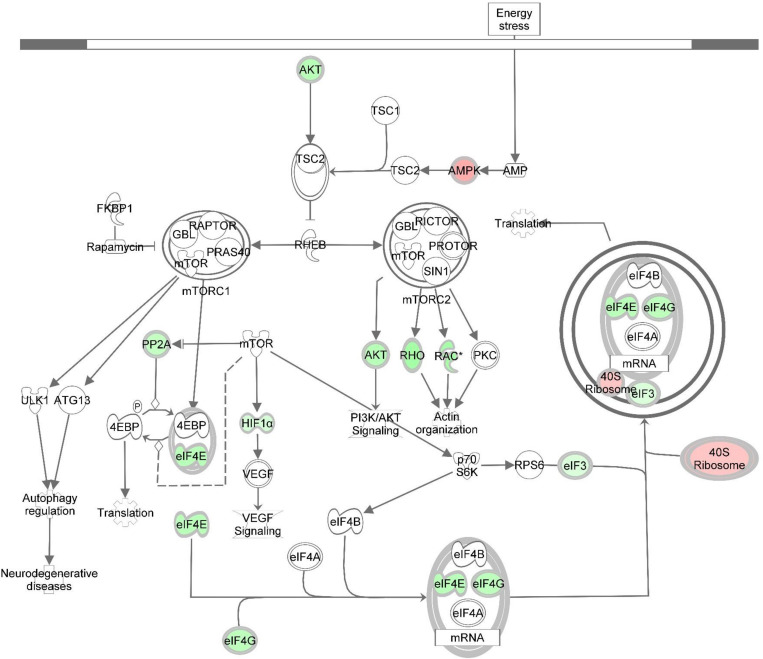
Knockdown of *DAP* alters gene expression of the mTOR signaling pathway during proliferation of turkey satellite cells. Intermolecular connections between genes are as defined by IPA analysis. *FDR < 0.05. 4EBP, 4E binding protein; ATG13, autophagy related 13; eIF3, eukaryotic translation initiation factor 3; eIF4A, eukaryotic translation initiation factor 4A; eIF4B, eukaryotic translation initiation factor 4B; eIF4G, eukaryotic translation initiation factor 4G; FKBP1, FK506 binding protein 1A; GBL, G protein beta subunit like; HIF1α = hypoxia inducible factor 1 alpha subunit; mTOR, mechanistic target of rapamycin; mTORC1, mechanistic target of rapamycin complex 1; mTORC2, mechanistic target of rapamycin complex 2; p70S6K, p70S6 kinase; PI3K, phosphoinositide 3-kinase; PKC, protein kinase C; PP2A, protein phosphatase 2; catalytic subunit, alpha isozyme; PRAS40, proline-rich Akt substrate 40; RAC, Ras-related C3 botulinum toxin substrate; Rheb, Ras homolog enriched in brain; Rho, Rhodopsin; RPS6, ribosomal protein S6; TSC1, tuberous sclerosis protein 1; TSC2, tuberous sclerosis protein 2; VEGF, vascular endothelial growth factor. Red highlighting indicates up-regulated genes; green highlighting indicates down-regulated genes. This pathway was generated through the use of Ingenuitay Pathway Analysis (QIAGEN, Inc. https://digitalinsights.qiagen.com/products-overview/discovery-insights-portfolio/analysis-and-visualization/qiagen-ipa/).

**FIGURE 6:**
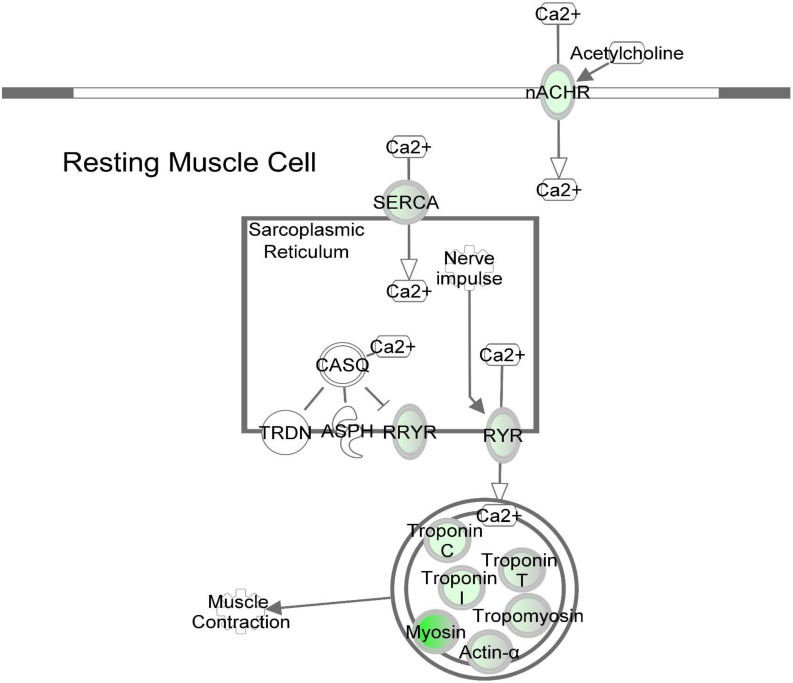
*DAP* knockdown alters expression of calcium signaling genes during differentiation of turkey satellite cells. Intermolecular connections between genes are as defined by IPA analysis. *FDR < 0.05, **FDR < 0.01, and ***FDR < 0.001. ASPH, aspartate beta-hydroxylase; CASQ, calsequestrin; DHPR, dihydropyridine receptor; nACHR, nicotinic cholinergic receptor gamma subunit; RYR, ryanodine receptor; RRYR, RYR interaction network; SERCA, sarcoplasmic/endoplasmic reticulum Ca^2+^-ATPase; TRDN, Triadin. Symbols are defined as in Figure 4. Red highlighting indicates up-regulated genes; green highlighting indicates down-regulated genes. This pathway was generated through the use of Ingenuitay Pathway Analysis (QIAGEN, Inc. https://digitalinsights.qiagen.com/products- overview/discovery-insights-portfolio/analysis-and-visualization/qiagen-ipa/).

DE transcripts (FDR < 0.05) were subjected to IPA analysis using their official gene names and canonical pathways considered in interpreting results. The most affected canonical pathways within *DAP*-knockdown proliferating and differentiation satellite cells are shown in Table 2. Knockdown of *DAP* in turkey satellite cells during proliferation altered expression of several eukaryotic initiation factor (eIF) genes as well as mTOR signaling genes (Figure 5). During differentiation, the calcium signaling pathway (Table 2, Figure 6) was the primary canonical pathway altered by *DAP* knockdown. In addition, actin cytoskeleton signaling, integrin-linked kinase (ILK) signaling, regulation of actin-based motility by Rho and protein kinase A are also revealed by IPA as affected canonical pathways (Table 2). IPA calculates activation (*z*) scores that infer the activation states of genes with predicted functional interactions within molecular networks. All of these enriched pathways exhibited a negative *z*-score, indicating a predicted inactivation. The impact of differential expression on molecular functions include aspects of cell morphology, cellular organization, and cellular maintenance (Table 3). Many of the genes showing significant changes in expression (FDR < 0.05) are translated into proteins comprising the myofibrillar architecture including myosin, actin, tropomyosin, the troponin subunits, nebulin, and titin corresponding with the observed failure to form myotubes in our previous study on *DAP*-knockdown in satellite cells (Velleman et al., 2012).

**TABLE 2:** Top five canonical pathways affected by *DAP* knockdown in proliferating and differentiating satellite cells.

**Stage**	**Canonical pathways**	***p*-value**
Proliferation	EIF2 signaling	9.68 × 10^–11^
	Protein ubiquitination pathway	9.98 × 10^–8^
	Sirtuin signaling pathway	2.69 × 10^–7^
	Regulation of eIF4 and p70S6K signaling	1.56 × 10^–6^
	mTOR signaling	1.62 × 10^–6^
Differentiation	Calcium signaling	7.40 × 10^–15^
	Actin cytoskeleton signaling	3.14 × 10^–8^
	ILK signaling	4.99 × 10^–7^
	Regulation of actin-based motility by Rho	1.05 × 10^–6^
	Protein kinase A signaling	1.07 × 10^–6^

**TABLE 3:** Top five altered molecular and cellular functions affected by *DAP* knockdown in proliferating and differentiating satellite cells.

**Stage**	**Altered functions**	***p*-value range**
Proliferation	Protein synthesis	7.64 × 10^–6^–6.36 × 10^–15^
	Cell death and survival	8.69 × 10^–6^–2.59 × 10^–9^
	Cellular assembly and organization	4.35 × 10^–8^–4.35 × 10^–8^
	DNA replication, recombination, and repair	4.35 × 10^–8^–4.35 × 10^–8^
	Cellular movement	9.06 × 10^–7^–1.11 × 10^–7^
Differentiation	Cell morphology	3.04 × 10^–4^–5.88 × 10^–19^
	Cellular assembly and organization	3.07 × 10^–4^–2.02 × 10^–16^
	Cellular function and maintenance	2.77 × 10^–4^–8.89 × 10^–16^
	Cellular development	3.37 × 10^–4^–3.69 × 10^–15^
	Cellular growth and proliferation	3.37 × 10^–4^–3.69 × 10^–15^

Pathway analysis of the 80 DE loci common to both proliferation and differentiation knockdown experiments showed the most affected canonical pathways include nitric oxide signaling in the cardiovascular system, neuregulin signaling, mTOR signaling, glucocorticoid receptor signaling and unfolded protein response (Table 4). In an IPA comparison analysis, the largest activation score was observed for nitric oxide signaling in the cardiovascular system (activated, *z* = 1.34, *p* = 8.79 × 10^–6^). This result is based primarily on expression of heatshock proteins (*HSP90B1, HSP90AA1*, and *HSP25*), *AKT1*, and AMPK (*PRKAA1*).

**TABLE 4:** Top five canonical pathways with shared genes affected by *DAP* knockdown in proliferating and differentiating satellite cells based on *p*-value.

**Canonical pathways**	***p*-value**
Nitric oxide signaling in the cardiovascular system	8.79 × 10^–6^
Neuregulin signaling	1.56 × 10^–4^
mTOR signaling	3.14 × 10^–4^
Glucocorticoid receptor signaling	3.63 × 10^–4^
Unfolded protein response	5.30 × 10^–4^

## Discussion

Myocyte proliferation and differentiation are the result of tightly regulated, temporal changes in gene expression (Buckingham et al., 2003). The processes are not only required for skeletal muscle growth and development, but also for muscle regeneration to maintain integrity of mature adult skeletal muscle. Biological pathways associated with muscle development have been intensively studied; however, many genes involved in this process are as of yet unidentified or their specific roles are not fully elucidated. In a previous transcriptome analysis to characterize temporal changes in gene expression, our group found that *DAP* transcription was highest in embryonic *pectoralis major* muscle undergoing hyperplasia, followed by a substantial progressive decline in expression as muscle development shifted to hypertrophy in the 1-day-old hatchling (Sporer et al., 2011b). Transcript levels of *DAP* continued to decline between 1 day and 16 weeks of age. Subsequently, we performed siRNA knockdown experiments to elucidate the role of *DAP* in proliferating and differentiating of *pectoralis major* muscle satellite cells. Velleman et al. (2012) observed that *DAP* knockdown resulted in substantial inhibition of both satellite cell proliferation and differentiation *in vitro*. Similar results were observed in chicken satellite cell cultures (Shin et al., 2013a). Conversely, overexpression of *DAP* in chicken satellite cells resulted in increased myotube diameter and higher rates of proliferation and differentiation, further suggesting a role for DAP in muscle development based on results from this cell culture model.

The current study was designed to elucidate the molecular mechanisms by which DAP mediates skeletal muscle growth and development. Transcriptomes of *DAP*-knockdown turkey *pectoralis major* muscle satellite cells were profiled during proliferating and differentiating states against those of non-treated control cells. To our knowledge, these results provide the first evidence that reduced *DAP* expression dramatically alters global gene expression in both proliferating and differentiating turkey satellite cells.

Knockdown of *DAP* in proliferating satellite cells, as demonstrated by microarray analysis, showed expression of this gene was reduced approximately three-fold compared to the control cells. In complementary experiments, an approximate five-fold reduction of *DAP* was observed by qPCR (Figure 3). However, in the differentiating cells, no significant difference in *DAP* abundance was observed between treated cells and controls by either microarray or qPCR studies. The absence of *DAP* differential expression during differentiation was likely the result of the transient transfection system used for these experiments. As described previously (Velleman et al., 2012), satellite cells were first transfected with siRNA, grown to 65% confluence (about 72 h), and then induced to differentiate over a period of 48 h. The half-life of *DAP* transcripts remains unknown and the treatment conditions may have provided enough time for the cells to transcribe sufficient levels of *DAP* such that levels were not significantly different between the knockdown and control cells by the 48 h differentiation timepoint. It is also important to note that changes in transcript abundance due to siRNA transfection do not necessarily result in changes in protein levels.

### Proliferation

In proliferating turkey *pectoralis major* muscle satellite cells, a majority of the affected genes were down-regulated compared to the control. The microarray data were corroborated with qPCR, with all of the examined genes showing similar overall trend in fold change. Pathway analysis (Table 2) suggested that knockdown of *DAP* in proliferating turkey satellite cells altered the cellular signals regulating post-transcriptional processes, in large part, through the modification of eIFs and protein turnover. The deviation of such canonical signals would affect cellular activities, particularly protein synthesis, and eventually in extreme cases, lead to cell death (Table 3).

The mTOR signaling pathway was one of the most significantly affected canonical pathways in response to *DAP* knockdown. The mTOR enzyme is a serine-threonine protein kinase that regulates cell growth, proliferation, and development in response to nutritional and environmental cues (Hay and Sonenberg, 2004; Wullschleger et al., 2006). This pathway is regulated in part by DAP which is a substrate for phosphorylation (Koren et al., 2010). Studies suggest that mTOR is important in regulating the ultimate growth and size of skeletal muscle (Bodine et al., 2001; Pallafacchina et al., 2002; Ohanna et al., 2005). This is due, in large part, to control of the underlying mechanisms of protein synthesis (Wullschleger et al., 2006; Zanchi and Lancha, 2008; Sandri, 2010).

In this study, several eIFs, essential for initiation of eukaryotic translation (i.e., protein synthesis), showed altered gene expression as a result of *DAP* knockdown in proliferating satellite cells (Supplementary Table S2 and Figure 4). Fold change of nine initiation factors affected by *DAP* knockdown (*eIF2S1*, *eIF3A*, *eIF3D*, *eIF3E*, *eIF3L*, *eIF3J*, *eIF4E*, *eIF4G2*, and *eIF5*) ranged from 0.22 to 29.22. Altered expression of these initiation factors can affect satellite cell proliferation and differentiation (Ohanna et al., 2005; Wullschleger et al., 2006). For example, *eIF4E* was down-regulated more than fourfold (FDR < 0.05) in proliferating turkey satellite cells upon *DAP* knockdown. The translational protein eIF4E recognizes and binds to the 5′cap of mRNA, aiding in the recruitment of mRNA to the ribosomal complex (Aitken and Lorsch, 2012). A second eIF (*eIF4G2*), also commonly known as *p97* or *DAP5*, was down-regulated nearly fourfold (FDR < 0.05). Reduced expression of *eIF4G2* by siRNA in HEK293 cells led to decreased overall translation rate and inhibition of cell proliferation (Lee and McCormick, 2006). Other eIFs affected by DAP knockdown include the *eIF3*s that are necessary for formation of the pre-initiation complex with the 40S ribosomal subunit during translational initiation (Aitken and Lorsch, 2012).

In the present study, differential expression of several eiF3 subunits was observed with *DAP* knockdown (FDR < 0.05). One subunit (*eIF3D*) was down-regulated more than twofold while, the other four, including *eIF3A*, *eIF3E*, *eIF3J*, and *eIF3L*, were up-regulated. Among these four, *eIF3E* was the most DE in the proliferating cells with FC > 29-fold. Differential expression changes of *eIF3A* and *eIF3L* were comparable (∼fivefold) whereas *eIF3J* was increased more than ninefold. Because all other *eIFs* showed a decrease in expression upon knockdown of *DAP*, it is proposed that expression of the up-regulated *eIF3s* may be a compensatory mechanism for loss of translational control at early stages during the translation initiation process. Consistent with our findings, yeast cells with reduced expression of eIFs display altered cell cycle profile with an accumulation of the cells in G1 phase (Yu et al., 2006).

Another critical gene significantly altered by *DAP* knockdown during proliferation was *AKT1*, down-regulated >4-fold relative to control cells (FDR < 0.05). This gene encodes the AKT1 protein (also termed protein kinase B) that plays important roles in cell metabolism, proliferation, and growth. AKT1 exerts an anti-apoptotic role in cell survival, and mediates cell growth through the mTOR pathway (Manning and Cantley, 2007; Sandri, 2010). In mice, targeted knockdown of *AKT1* in C2 myoblasts blocked differentiation, although with no apparent effect on cell proliferation (Rotwein and Wilson, 2009). Conversely, Héron-Milhavet et al. (2006) found AKT1 to play a critical role in proliferation of C2.7 myoblasts and 3T3 fibroblasts. Goncalves et al. (2010) showed that Akt-knockout mice had reduced muscle mass, grip strength, and contractile force. Thus, our results indicating reduced *AKT1* expression associated with the *DAP* knockdown are consistent with impaired proliferation and development. In addition, as noted by Sandri (2010) AKT1 inhibits autophagosome formation and lysosome-directed protein degradation. Thus, it is reasonable to hypothesize that the degree of suppression of *AKT1* expression in the *DAP* knockdown cells may result in increased degradation of muscle proteins due to the absence of the inhibitory effect of AKT1.

The current findings highlight the impaired protein biosynthesis, particularly via modified translation initiation and mTOR signaling, in the *DAP*-knockdown satellite cells. Such molecular alteration might in turn restrict progression of the cells to initiate a new round of cell division, hence diminishing cell proliferation in the knockdown cells (Polymenis and Aramayo, 2015).

### Differentiation

Based on pathway analysis, the calcium signaling pathway was the top altered canonical pathway reported by IPA (Table 2 and Figure 6). A total of 13 genes in this pathway displayed differential expression upon *DAP* knockdown with expression ranging from 34-fold decrease to 2.5-fold increase. Proteins in the calcium signaling pathway include SR calcium regulators that are responsible for calcium uptake and release, and myofibrillar proteins that respond to changes in [Ca^2+^] to effect muscle contraction. Of the SR calcium regulators, the voltage dependent calcium channel subunit gamma 1 (*CACNG1*) and both isoforms of avian ryanodine receptors, i.e., *RYR1* and *RYR3* were down-regulated approximately 28-fold, 2-fold, and 6-fold, respectively. The *CACNG1* gene encodes the dihydropyridine receptor (DHPR) gamma subunit, one of the five subunits of the L-type calcium channel (Rossi and Dirksen, 2006). The DHPR is located in the transverse tubule and associates with RYR1 to elicit calcium release from the SR into the sarcoplasm (Rossi and Dirksen, 2006; Huang et al., 2011). The gamma subunit of the DHPR, in particular, may act as a calcium antagonist during times of cell stress (Andronache et al., 2007). Both RYR1 and RYR3 are calcium channel proteins that open upon activation to allow diffusion of Ca^2+^ from SR lumen to the cytosol. The sarco/endoplasmic reticulum-Ca^2+^ ATPase is responsible for reducing cytosolic Ca^2+^ by active transport, coupled with the hydrolysis of ATP, back into the SR (Rossi and Dirksen, 2006). The SERCA gene *ATP2A2* showed nearly threefold down-regulation (FDR < 0.05) upon *DAP* knockdown. Although SERCA2 is more commonly associated with slow-twitch and cardiac muscle, SERCA2a, the more prominent of the SERCA2 isoforms, has been detected in fast-twitch skeletal muscle and neonatal muscle in humans (Zarain-Herzberg and Alvarez-Fernandez, 2002). Proteins that comprise most of the mass of the muscle myofibril were also downregulated, including the structural proteins titin and nebulin, the contractile proteins myosin and actin, and the Ca^2+^-sensitive regulatory proteins tropomyosin and the three troponin subunits. In addition, obscurin, which may play a role in initiating myofibril assembly (Carlsson et al., 2008), was also downregulated, potentially reducing myotube development observed in the knockdown experiments (Velleman et al., 2012).

It is widely recognized that Ca^2+^ acts as a second messenger responsible for regulating cascades of signal transduction. Differential expression of the crucial Ca^2+^-regulatory proteins responsible for release and uptake of cytosolic Ca^2^ in differentiating satellite cells suggests aberrant cytosolic [Ca^2+^] homeostasis with profound downstream consequences for cellular development. In myogenesis, precise control of [Ca^2+^] is required for activating muscle-specific transcription factors that regulate the fusion of myoblasts to form multinucleated myotubes (Fu et al., 2015). Dysregulated [Ca^2+^] in the *DAP*-knockdown cells may interfere with numerous cellular processes including the calcium-mediated pathways that are critical for myotube formation.

The other top altered canonical pathways (Table 2) function in a coordinated manner to regulate cytoskeletal reorganization largely required for cell differentiation. The cytoskeleton, recognized as a highly dynamic scaffold, comprises different molecules, e.g., actin and tubulin, arranged into filaments distributed throughout the cytoplasm. Reorganization of such structures induced by extracellular stimuli, activates intracellular signaling, including Rho-family small GTPases through the links with extracellular matrix adhesion molecules such as integrins and syndecans (Shin et al., 2013b; Yen et al., 2014; Ambriz et al., 2018). In this study, two main cytoskeletal proteins, including actin and tubulin, were substantially down-regulated. Within the *DAP*-knockdown cells, decreased transcripts include two loci corresponding to alpha actin (*ACTA1*, FC = −19 and −12), cardiac actin (*ACTC1*, FC = −16) and three loci of alpha tubulin (*LOC100545462*, FC ranging from −5.5 to −9.2). In addition, expression of integrin beta-1 (*ITGB1*), an integrin complex subunit, and filamin (*FLN2*), that serves as a linking protein between two actin filaments, were decreased 4.9-fold and 3.5-fold, respectively. In contrast, syndecan-2 (*SDC2*) increased 4.3-fold. Two loci of the non-regulatory myosin light chain (*MYL1*) were the most highly down-regulated at >143-fold and >59-fold. Structurally and functionally different from myosin heavy chain, myosin light chain resides in the neck region of myosin and interacts with Rho A effectors, hence mediating assembly of actomyosin complex. With altered cytoskeleton signaling, the formation of multinucleated myotubes could be disrupted, thus preventing the differentiation of the knockdown cells.

There were 80 DE loci common to both proliferation and differentiation. Of the top five canonical pathways affected (Table 4), the most dramatically affected pathway, Nitric Oxide Signaling in the Cardiovascular System, is of particular interest. The DE genes within this pathway include upregulation of the heat shock proteins (*HSP90AA1* and *HSP90B*), the catalytic subunit of *AMPK* (*PRKAA1*), and downregulation of *AKT1*. It is well established that AKT1 plays a critical role in regulating proliferation, differentiation, size, and viability of muscle cells in part through changes in phosphorylation status (Yun and Matts, 2005). The protein HSP90 plays multiple roles in the developing muscle cell. Like most heat shock proteins, it is involved in protein folding and in refolding of denatured proteins (Sato et al., 2000). In addition, HSP90 binds to AKT1 and maintains a balanced phosphorylation state of AKT1 by reducing dephosphorylation through protein phosphatase 2A (PP2A) (Sato et al., 2000; Yun and Matts, 2005). The substantial downregulation of *AKT1* coupled with upregulation of *HSP90AA1* and *HSP90B* could modulate this balance. Moreover, HSP90 is a target of *S*-nitrosylation that results in loss of its ATP-dependent protein folding activity (Martínez-Ruiz et al., 2005), and could result in accumulation of misfolded proteins accelerating autophagy or apoptosis. Finally, the upregulation of *PRKAA1* is associated with decreased protein synthesis through phosphorylation of mTOR and tuberous sclerosis complex 2 (TSC2), thereby increasing autophagy (Thompson, 2018). AMPK also phosphorylates key regulators of autophagy including ULK1, ATG9, and beclin-1.

Early studies of DAP in HeLa cells suggest it is an important positive regulator of cytokine-mediated apoptosis (Deiss et al., 1995). Subsequently, Koren et al. (2010) showed that DAP is phosphorylated under nutrient-rich conditions but functionally silent under starvation conditions where DAP is dephosphorylated and acts as a suppressor of autophagy. The process of autophagy is implicated in skeletal muscle development and renewal as a method for efficiently recycling and removing proteins from cells (Sandri, 2010). Ca^2+^, acting as a second messenger, regulates the processes of autophagy and apoptosis through complex spatiotemporal mechanisms that may either promote or inhibit these pathways (Harr and Distelhorst, 2010; Sun et al., 2016). Increased or decreased intracellular [Ca^2+^] may disrupt regulation of these pathways, leading to altered cellular development. The results of the current study suggest that DAP plays a central role in turkey muscle growth and development, and perhaps in other cell types, by regulating autophagy as well as calcium signaling and protein translation.

In conclusion, this study demonstrated global changes in the transcriptome following knockdown of *DAP* in developing turkey satellite cells. The findings underline the essential roles of DAP in proliferation, potentially through the transduction signals associated with translation initiation and mTOR signaling. During differentiation, DAP knockdown altered the biological pathways responsible for controlling [Ca^2+^] and cytoskeleton organization. Future studies will focus on mechanisms by which changes in *DAP* expression, and consequent changes in the level of cellular DAP protein, alter cellular homeostasis.

## Data Availability Statement

Raw Cy5 and Cy3 intensities, M-value, and LOESS-normalized log2 average intensities (A) were submitted with original GPR files to the NCBI GEO (platform accession: GPL9788, series accession: GSE35660).

## Author Contributions

GS, SV, and KR designed the experiments and acquired the funding for this project. SV performed the cell culture work including *DAP* knockdown experiments. KH and KS isolated RNA and performed the microarray and qPCR experiments. RT analyzed the raw microarray data. KR annotated the microarray oligonucleotides and conducted the IPA analysis. KH made the initial draft of the manuscript with major revisions provided by YM, KR, SV, and GS. All authors read, revised, and approved the final manuscript.

## Conflict of Interest

The authors declare that the research was conducted in the absence of any commercial or financial relationships that could be construed as a potential conflict of interest.
