# Supplementary material for: Knockdown of Death-Associated Protein Expression Induces Global Transcriptome Changes in Proliferating and Differentiating Muscle Satellite Cells
Source: Front Physiol. 2020 Aug 14;11:1036. doi: 10.3389/fphys.2020.01036 (PMC7457014; doi:10.3389/fphys.2020.01036)
Supplement: Supplementary file 1 [file Table_1.DOCX]

**Table S1.** Primer sequences for qPCR.

| **Gene Symbol** | **Sequence** | **Amp Length**  **(bp)** | **T_m_ (⁰C)** |
| --- | --- | --- | --- |
| *ACTA1* | 5’-TGGGATCCATGAGACAACTTACA-3’ (Forward)  3’- ACAGGTCCTTCCTGATGTCAATG-5’ (Reverse) | 78 | 65 |
| *AKT1* | 5’-GCCAGTGGAACCGCTTAATG-3’ (Forward)  3’- TCCCCAAAATGCAGTCTGTTT-5’ (Reverse) | 69 | 77 |
| *ANXA6* | 5’-CAGGAGTTCGTCAAAATGACCAA-3’ (Forward)  3’- CGTACGATGGCCACAAAGG-5’ (Reverse) | 95 | 83 |
| *ATP2A2* | 5’-ATGTGTGCAAATCGACCTGTTT-3’ (Forward)  3’- GGAGTCGTTTGCTGCTGTCTAA-5’ (Reverse) | 123 | 76 |
| *BHMT* | 5’-CTGCTTCAGGCAGGCCATA-3’ (Forward)  3’- GGTCACTCCCCAGCCATCT-5’ (Reverse) | 59 | 82 |
| *CACNG1* | 5’-AAGGATGATCGACAGCAAGGA-3’ (Forward)  3’-CAGGCGAAGGACCAGGAGTA-5’ (Reverse) | 63 | 82 |
| *DAP* | 5’-TGGGCAGCTCTACTCTATGTGTTC-3’ (Forward)  3’-CAAGAGCAGTTGTTTCTAACTCAAAAG-5’ (Reverse) | 79 | 78 |
| *ENO1* | 5’-CACCAGCTCTGATTTGTTACTTGTAAT-3’ (Forward)  3’-ACACAAAACCCCACTGAGACTGT-5’ (Reverse) | 69 | 77 |
| *FTH1* | 5’-TGGCATGGCAGAGTACCTGTT-3’ (Forward)  3’-GGCAAGCCTTCAGCTGTCA-5’ (Reverse) | 64 | 83 |
| *IMP2C* | 5’-AGCCCCTGTCCAGTTGTAACC-3’ (Forward)  3’-GAGGGCTGAGAGCATGTGTGT-5’ (Reverse) | 60 | 82 |
| *PCOLCE2* | 5’-ATCATGGGCCAGGTAGAAGAAG-3’ (Forward)  3’-CATGACAAAGCTGTTCGGAAAG-5’ (Reverse) | 60 | 79 |
| *TNNC2* | 5’-CTGAGCTTTTCCTTGACTCTGACA-3’ (Forward)  3’-TTCAGCCTTTCCCCTTTTAATG-5’ (Reverse) | 68 | 77 |
| *TNNT2* | 5’-GGCTCAGCCATCAGATGCA-3’ (Forward)  3’-CAGCAGAGCCCTGGCATAG-5’ (Reverse) | 54 | 85 |
| *TPM1* | 5’-AGACATCTTCATCGGGTTGGA-3’ (Forward)  3’-GACAGGACAGAGAACCATGAAACA-5’ (Reverse) | 70 | 77 |
| *UNC5B* | 5’-TGACCGCGTCTTCAACATCTT-3’ (Forward)  3’-CCGGTTTGCTCTTCATGAACTT-5’ (Reverse) | 71 | 81 |
